# Examining the relationship between suicide ideation frequency and intergenerational acculturative conflict between Mexican descent college students and their caregivers using the interpersonal theory of suicide

**DOI:** 10.1111/sltb.13067

**Published:** 2024-02-27

**Authors:** Jocelyn I. Meza, Brandy Piña‐Watson, Daisy Lopez, Gisel Suarez Bonilla, Maria R. Sanchez, Gabriela Manzo, Aundrea Garcia

**Affiliations:** ^1^ Department of Psychiatry and Biobehavioral Sciences University of California Los Angeles California USA; ^2^ Department of Psychological Sciences Texas Tech University Lubbock Texas USA

**Keywords:** college, Latine, sociocultural, suicide

## Abstract

**Introduction:**

Suicide is the third leading cause of death among US young adults, with significant racial/ethnic disparities related to the risk for suicide among Latine young adults. Despite the elevated risk for suicide, culturally relevant risk factors are not well‐known. Intergenerational acculturative conflict (IAC) among Latine youth is a sociocultural factor associated with suicide ideation.

**Method:**

Although widely cited, the interpersonal theory of suicide (IPTS) lacks consistent support among Latine groups. The following cross‐sectional study examined relationships between IAC categories (cultural preference, autonomy, and dating/staying out late), IPTS risk factors (i.e., thwarted belongingness and perceived burdensomeness), and suicide ideation frequency among 376 Mexican descent college students sampled using participant pools and snowball sampling (73.7% female: M_age_ = 19.88).

**Results:**

Mediation analyses supported the hypotheses that IPTS risk factors partially explained the links between IAC categories and suicide ideation frequency.

**Conclusions:**

These findings advance our understanding of how sociocultural constructs, such as IAC, influence the IPTS and future advancements in culturally responsive treatments for suicide.

## INTRODUCTION

The interpersonal theory of suicide (IPTS) was developed to understand the precipitants of suicide ideation and attempts while considering the importance of social relationships (Joiner, 2005). IPTS posits that the co‐occurrence of two factors results in suicide ideation—perceived burdensomeness (PB) and thwarted belongingness (TB). PB is when an individual believes they are a burden to others and their death would be valued more than their life. PB comprises one's perception of liability and self‐hate (Aceves & Piña‐Watson, 2021; Chu et al., 2017). TB comprises loneliness and the absence of reciprocal care (Aceves & Piña‐Watson, 2021; Chu et al., 2017). When an individual experiences TB, they believe they are not an essential social group member leading to an increased perception of social isolation and disconnectedness (Acosta et al., 2017). Chu et al. (2017) conducted a meta‐analysis using 122 distinct unpublished and published samples, concluding that the IPTS is a well‐documented theory supported by various studies.

Despite ample evidence supporting IPTS, it has been primarily examined among non‐Latine White groups, highlighting a significant gap in our understanding. The following studies are a sample of the limited literature found for Latine groups. Garza and Pettit (2010) found greater PB to predict greater suicide ideation among Mexican American women. However, researchers did not test TB in this study. Gulbas et al. (2019) used qualitative methods to find greater TB and PB among 20 out of 30 Latine adolescents who had attempted suicide and no TB or PB among 22 of 30 adolescents who had no history of suicidal behaviors. Finally, Acosta et al. (2017) tested the predictive ability of TB, PB, and ethnicity (White and Hispanic/Latine) on levels of suicidal desire among undergraduate college students. When examining the association between TB, PB and suicidal desire among the entire sample, Acosta and colleagues found that greater TB and PB was associated with greater suicide desire. In addition, results indicated that Latine individuals reported less PB and suicide desire than their White counterparts, while no significant differences between ethnic groups were found in endorsements of TB (Acosta et al., 2017). Interestingly, when ethnicity was added as a moderator, Acosta et al. (2017) found that the greatest suicide desire was found among White individuals who reported greater PB and TB.

Across all ages, suicide is the 12th leading cause of death in the United States, and among adolescents aged 13–17, suicide is the second leading cause of death (CDC, 2021). Within emerging adults aged 18–24, suicide is still the third leading cause of death (CDC, 2021). Therefore, despite adolescence being the highest risk period for the onset of suicide ideation and suicide attempts, the risk for suicide remains high well into young adulthood (Glenn et al., 2017; Nock et al., 2012). During the past decade, young Latina adults have experienced increased suicide rates (Silva & Van Orden, 2018). Approximately 8.5% of Latine college students also experience suicide ideation (Goodwill & Zhou, 2020). Emerging adulthood is a risky period for suicide among minoritized college students, such as Mexican descent students, due to their developing identities, the exacerbated intercultural interactions on college campuses, and certain cultural expectations at home (Oakey‐Frost et al., 2021). Given that individuals of Mexican descent are the largest Latine ethnic group in the United States (Noe‐Bustamante, 2019; Saenz, 2010), understanding factors associated with these mental health disparities is pivotal. Examining suicide ideation, a key risk indicator of suicide, and the IPTS among emerging adult Mexican descent college students would contribute significantly to the literature.

### Intergenerational acculturative conflict

Acculturation is a dual process that occurs when an individual joins a different culture and accepts cultural values from the majority culture while maintaining cultural values from their heritage culture (Schwartz et al., 2010). This bi‐dimensional process occurs at the behavioral, cognitive, and affective levels, with each person acculturating and enculturating at different rates. Intergenerational acculturative gaps occur when parents and their children acculturate at differing rates (Birman & Poff, 2011). The acculturation gap hypothesis postulates that these differences are associated with increased intergenerational acculturative conflict (IAC) between children and their parents, which is associated with poorer outcomes for the youth (Lui, 2015; Manzo et al., 2022; Meza et al., 2022). For example, among Mexican descent adolescents and emerging adults, increased levels of IAC have been related to several mental health outcomes such as depressive symptoms, distress, and suicide ideation (Hernández et al., 2010; Piña‐Watson et al., 2015, 2019, 2020). However, there is no research on how IAC leads to greater suicide ideation or if the link between IAC and suicide ideation fits with prominent theories of suicide.

Latine culture often places a high value on the family system and requires that individuals place family functioning above the self. This ideology is often cited as the construct of familism (Aceves & Piña‐Watson, 2021). Given these familism values, it is possible that greater IAC among Latine individuals and their caregivers contributes to feelings of TB and/or PB and can activate greater suicide ideation. Indeed, Castillo et al. (2007) found that differing acculturation levels among a racially diverse sample increased IAC and was associated with greater endorsement by children and caregivers of feeling disconnected from one another. Additionally, research has found that individuals who experience family conflict in general are more likely to develop perceptions of burdensomeness and decreased feelings of belonging (Van Orden et al., 2010). Despite these associations, IAC and perceptions of PB and TB have yet to be examined in a sample of young adult Latine college students.

Most research on suicide risk and IAC has focused on adolescents (Querdasi & Bacio, 2021) and neglected to examine the different types of IAC and their link to mental health outcomes. Examining the different types of IAC is imperative given current findings from factor analyses about different types of IAC (i.e., autonomy conflicts, conflicts over preferred culture, and dating/being out late conflicts) and the types of IAC being differentially associated with young adult adjustment outcomes (Basáñez et al., 2014). Latine college students may experience greater IAC given the identity development, intercultural interactions, acculturative stress, and discrimination that occurs on college campuses, as well as discrepancies with students' cultural expectations at home (Oakey‐Frost et al., 2021). Furthermore, a meta‐analysis examining studies on IAC gaps found that the relationship between family conflict and poorer mental health was more significant among young adults than adolescents (Lui, 2015). In a recent study of Latine emerging adults, IAC predicted higher depression symptoms, although the different types of IAC and suicide risk outcomes were not examined (Rahman et al., 2023). Therefore, there is a significant need to examine the different types of IAC and their association with the IPTS among emerging adult Mexican descent college students.

### Current study

The current study aimed to examine if different types of IAC between Mexican descent college students and their male and female caregivers predicted suicide ideation frequency, and whether PB and TB mediated this link. We hypothesized that:

Hypothesis 1 Higher ratings of IAC (i.e., autonomy conflicts, cultural preference conflicts, and dating/being out late conflicts) with female caregiver will be associated with greater suicide ideation frequency.

Hypothesis 2 Higher ratings of IAC (i.e., autonomy conflicts, cultural preference conflicts, and dating/being out late conflicts) with male caregiver will be associated with greater suicide ideation frequency.

Hypothesis 3 PB and TB will help explain the links between male and female caregiver IAC and suicide ideation frequency.

## METHODS

### Participants

Participants were 376 Mexican descent emerging adult college students (73.7% women; 20.7% men, 2.7% transgender, and 3% gender non‐binary) enrolled in Hispanic serving institutions (HSIs) in West Texas and colleges nationwide. Students who were enrolled in HSIs and received course credit were 53.7% of the sample (71.78% women, 26.24% men, and 0.01% gender non‐binary), while 46.3% were students recruited via the snowball sampling method across the nation (75.86% women; 14.36% men, 0.06% transgender, and 0.02% gender non‐binary). Mexican descent was assessed by asking the following two questions: “Are you of Latina/o/x or Hispanic descent?” followed by “You indicated that you are Latina/o/x or Hispanic. Please select the country your family has lineage/heritage from in Latin America and select all that apply.” If participants indicated “Mexico” as a country their family has lineage/heritage from, they were considered Mexican descent and therefore eligible for the study. A total of 47 students were removed from the original sample because they did not pass one of the attention checks, and the original total sample was *n* = 423. The whole sample's ages ranged from 18 to 25 years (*M* = 19.88 years; *SD* = 1.76 years); more specifically, there were no significant differences across variables of interest between recruited students who received course credit (*M* = 19.15 years) and those who were recruited nationwide via the snowball sampling method (*M* = 20.73 years). Most of the students were first‐year college students (40.4%). Additionally, 1.3% of participants were first‐generation Mexican descent students (i.e., they were born outside of the United States and immigrated to the United States as adults over the age of 18), 2.9% were generation 1.5 (i.e., they were born outside of the United States and somewhat raised in their home country but moved to the United States before 18 years of age), 50.1% were second generation (i.e., had at least one foreign‐born parent), 34.9% were third‐generation (i.e., had two US native parents and at least one foreign‐born grandparent), and lastly, 10.7% were fourth‐generation. Regarding their living situation, 107 participants (28.5%) reported living with both female and male caregivers, 140 participants (37.3%) reported living alone, 53 participants (14.1%) said they lived with only their female caregiver, and 19 participants said they lived with their male caregiver only (5.1%), and 56 participants did not report on their caregiver living situation (14.9%). Socioeconomic status was measured using the students' perceptions of how other college students' family income compared to their family's income. The majority of students, 33%, reported having a lower income than others, 25.5% reported having a little lower income than others; 26.9% reported having the same income as others, 12% reported having a little more income than others, and 2.7% reported having somewhat more income than others.

### Procedures

This study was approved by the Institutional Review Board (IRB) of the second author's institution and was part of a more extensive study aimed at understanding risk and protective processes in mental health outcomes among Mexican descent college students in the United States. Upon approval by the IRB, data was collected in two methods. Participants were recruited from either an introductory psychology participant pool at the second author's institution (*n* = 202) or nationally through snowball sampling techniques (*n* = 174; e.g., emails, listservs, social media posts, word of mouth). Those who participated in the participant pool were compensated with course credit. Those who participated in the national sample were compensated with a $5 electronic gift card. Participation was voluntary; the participants' anonymous responses could not be linked to their identities. After participation, participants were given information on mental health resources at the local and/or national level. Participants had to self‐identify as Mexican/Mexican descent, live in the United States, be 18–25 years old, and be enrolled in college at the time of the study. Only those who met eligibility criteria were prompted to complete the rest of the survey and were eligible for compensation either monetarily or with course credit. Finally, three response validity indicators (e.g., Please check strongly agree) were incorporated throughout the survey to safeguard data quality (Chmielewski & Kucker, 2020). Participants who failed to respond correctly to these items were removed from the study.

### Measures

#### Intergenerational acculturative conflict

The 23‐item Acculturation Gap Conflict Inventory (AGCI; Basáñez et al., 2014) was used to examine types of IAC among young Mexican descent adults and their male and female caregivers. The young adults rate this measure to assess their perspective on present IAC with their caregiver. The AGCI includes three sub‐scales that assess the degree of conflict in autonomy, preferred culture, and dating/staying out late. For this study, each of these subscales' mean scores were used, with higher scores indicating more of the respective form of IAC. This measure was administered to the participant twice, one per primary caregiver, to obtain the level of IAC associated with the male and female caregivers separately. Based on whom individuals identified as their primary female caregiver/primary male caregiver, this information was auto‐populated using the “piped text” feature on Qualtrics so they could answer based on the respective caregiver. Each item was based on a 7‐point Likert‐type scale asking about the level of agreement with each statement ranging from 1 (*Strongly disagree*, *almost never*) to 7 (*Strongly agree*, *almost always*). Sample items include, “I wish that my parent would allow me to be more independent” (autonomy), “My parents wish I would practice customs of my culture more than I do” (preferred culture), and “I want to go on dates, but my parents do not want me to date” (dating). This measure demonstrated construct validity with the three factors, and each of the factors demonstrated acceptable internal consistency with a sample of Hispanic young adults (*α*_autonomy_ = 0.87; *α*_preferred culture_ = 0.87; *α*_dating_ = 0.84; Basáñez et al., 2014). There was good internal consistency in the present study (female caregiver: *α*_autonomy_ = 0.94; *α*_preferred culture_ = 0.91; *α*_dating_ = 0.90; male caregiver: *α*_autonomy_ = 0.94; *α*_preferred culture_ = 0.93; *α*_dating_ = 0.93).

#### Perceived burdensomeness and thwarted belongingness

The 15‐item Interpersonal Needs Questionnaire (INQ) measured PB (6 items) and TB (9 items; Van Orden et al., 2012). Each item on the scale ranges from 1 (*Not true at all for me*) to 7 (*Very true for me*). Mean scores were derived, with higher scores indicating higher PB and TB. Sample items include “These days, I think I am a burden on society” (PB) and “These days, I feel like I belong” (reverse coded for TB). Van Orden et al. (2012) supported convergent and discriminant construct validity. Aceves and Piña‐Watson (2021) used this scale with a sample of Mexican descent emerging adults and found acceptable reliability scores for both subscales (*α*_TB_ = 0.87; *α*_PB_ = 0.94). The alphas for the present study are *α*_TB_ = 0.73 and *α*_PB_ = 0.96. For the TB subscale, one item was removed (“These days I feel disconnected from other people”) due to its low alpha (0.68). Once removed, the internal consistency for this subscale was acceptable at the level of 0.73.

#### Suicide ideation frequency

Suicide ideation frequency was assessed from a single multiple‐choice item from the Suicide Behaviors Questionnaire‐Revised (SBQ‐R; Osman et al., 2001). The item included was “How often have you thought about killing yourself in the past year?” with the following answer options: (1) never, (2) rarely (1 time), (3) sometimes (2 times), (4) often (3–4 times) and (5) very often (5 or more times). A higher score in this item indicated greater frequency of suicide ideation in the past year. Given that prior research shows that increases in past year suicide ideation frequency is associated with future suicidal behaviors (Miranda et al., 2014), we used suicide ideation frequency as our outcome of interest in this study.

#### Demographics

A demographic questionnaire was used to gather participants' age, gender identity, college level, perceived socioeconomic status, generational status, and primary caregiver information. To assess gender identity, students were asked, “what is your gender identity?” with the following answer options: agender, androgyne, demigender, gender queer/fluid, man, questioning/unsure, trans man, trans woman, woman, an identity not listed or decline to state. To assess college level, participants were asked, “what is your college level?” with the following answer options: first year, sophomore, junior, senior and graduate student. Perceived socioeconomic status was assessed using a single‐item question asking participants, “compared to other students are your university, do you think your family's income is…” with the following answer options: somewhat less than most others, a little less than most others, same as most others, a little more than most others, and somewhat more than most others. Primary caregiver information was assessed via a multiple choice question of who they regarded as their primary caregiver, followed by a yes/no question asking if they were currently living with that caregiver. This question was asked twice, once for female caregiver and once for male caregivers. Generational status was asked using two questions: (1) “as far as you know, which of these people in your family were born outside of the United States? Check all that apply” with the following answer options: you, your mother, your father, all of your grandparents, some of your grandparents and all of these people were born in the United States, and (2) if students indicated that they were born outside the United States, they were asked at what age they arrived to the United States with a text option available.

### Data analysis plan

Statistical analyses were conducted using SPSS for Mac, Version 28. First, correlations were calculated to examine the bivariate relationship between all study variables. Then, means and standard deviations for all study variables were computed.

Linear regressions were used to test the first two hypotheses, examining the relationships between types of IAC with caregivers and suicide ideation frequency (Hypotheses 1 and 2). Suicide ideation frequency was regressed separately for the three IAC categories (autonomy conflicts, culture preference conflicts, dating/being out late conflicts) with female and male caregivers. All mean‐centered predictors and sociodemographic covariates (age, gender identity, generational status, currently living with their caregiver, and perceived socioeconomic status) were entered on the first step of regression analyses, with predictor variables of interest added in the second step.

Then, SPSS PROCESS model 4 was used to test the third Hypothesis of the pathways to suicide ideation severity. All models used bootstrapping, which allowed SPSS to create 10,000 random samples to generate 95% confidence intervals for significance testing. Six different models were calculated with TB and PB as potential factors explaining the link between IAC categories and suicide ideation severity. Three models tested the pathways between each IAC category with female caregivers and suicide ideation frequency, through TB and PB, and the other three models tested the pathways between each IAC category with male caregivers and suicide ideation frequency, through TB and PB. Several important background variables (i.e., age, gender identity, perceived socioeconomic status, currently living with their caregiver, and generational status) were included as covariates in all models. Very little data were missing for predictors, covariates, and the outcome variables of interest (i.e., 1%–2%), so none were imputed. The only exception was data for 15 participants' male caregiver conflict scales (3.9% missing) and 8 participants' female caregiver data (2.1% missing). However, these participants' data did not differ on any variables of interest (*p*s <0.05). Moreover, missing cases were not included in analyses for the models examining the caregiver conflict scales that were missing.

## RESULTS

### Descriptive analyses

Variables of interest were all normally distributed in this sample (i.e., skewness and kurtosis values <2), so no data transformations were conducted. A total of 33.6% (*n* = 126) of our sample reported having suicidal ideation in the past year, with varying frequencies reported such that 14.7% (*n* = 55) reported having suicidal ideation once in the last year, 10.7% (*n* = 40) reported having suicidal ideation twice in the last year, 4.5% (*n* = 17) reported having suicidal ideation 3–4 items in the last year, and 3.7% (*n* = 14) reported having suicidal ideation 5 or more times in the last year. Table 1 summarizes descriptive statistics and intercorrelations between all variables of interest. Mechanisms of interest, PB and TB, were positively and significantly correlated with each other, *r* = 0.510, *p* < 0.001. PB was significantly correlated with female caregiver autonomy conflicts: *r* = 0.427, *p* < 0.001; culture preference conflicts: *r* = 0.384, *p* < 0.001; dating/being out late conflicts: *r* = 0.385 *p* < 0.001; and suicidal ideation frequency: *r* = 0.340, *p* < 0.001. TB was also significantly correlated with female caregiver autonomy conflicts: *r* = 0.343, *p* < 0.001; culture preference conflicts: *r* = 0.302, *p* < 0.001; dating/being out late conflicts: *r* = 0.353 *p* < 0.001; and suicidal ideation frequency: *r* = 0.327, *p* < 0.001. Similar positive correlations emerged between proposed mechanisms and male caregiver conflict scales (see Table 1). All conflict scales with female caregivers were moderately to strongly correlated with conflict scales with male caregivers, *r*s = 0.398 to 0.725, *p*s <0.001.

**TABLE 1:** Correlations and descriptive statistics among measured variables.

Measured variables		1	2	3	4	5	6	7	8	9
1	Autonomy conflicts with FC	–								
2	Culture preference conflicts with FC	0.548*	–							
3	Dating/Being out late conflicts with FC	0.603*	0.566*	–						
4	Autonomy conflicts with MC	0.562*	0.426*	0.485*	–					
5	Culture preference conflicts with MC	0.398*	0.665*	0.475*	0.604*	–				
6	Dating/Being out late conflicts with MC	0.405*	0.481*	0.725*	0.629*	0.570*	–			
7	Perceived burdensomeness	0.427*	0.384*	0.385*	0.415*	0.430*	0.340*	–		
8	Thwarted belongingness	0.343*	0.302*	0.353*	0.304*	0.321*	0.296*	0.510*	–	
9	Suicide ideation frequency	0.300*	0.076	0.181*	0.309*	0.165*	0.239*	0.340*	0.327*	–
*N*		342	341	341	297	297	297	371	371	375
Range		1–7	1–7	1–7	1–7	1–7	1–7	0–6	0–6	1–5
*M* (SD)		3.55 (1.60)	2.35 (1.32)	3.10 (1.74)	3.53 (1.61)	2.45 (1.46)	3.27 (1.93)	0.91 (1.36)	2.06 (1.27)	1.65 (1.08)

Abbreviations: FC, female caregiver; MC, male caregiver.

* *p* < 0.01.

### Relationship between IAC categories and suicide ideation frequency

Regression analyses supported our first two Hypothesis that all IAC categories (autonomy conflicts, cultural preference conflicts, and dating/being out late conflicts) with female (Hypothesis 1) and male (Hypothesis 2) caregivers were associated with greater suicide ideation beyond young adults' age, gender identity, generational status, current living situation with their caregiver, and perceived socioeconomic status. The only non‐significant relationship was between culture preference conflicts with female caregivers and suicide ideation frequency (see Table 2).

**TABLE 2:** Relationship between female/male caregiver IAC categories and PB, TB, and suicide ideation frequency.

IAC categories	Perceived burdensomeness	Thwarted belongingness	Suicide ideation frequency
Autonomy conflicts with FC	*β* = 0.422 (0.257, 0.413), Δ*R* ^2^ = 0.173**	*β* = 0.342 (0.193, 0.358), Δ*R* ^2^ = 0.114**	*β* = 0.306 (0.141, 0.284), Δ*R* ^2^ = 0.091**
Culture preference conflicts with FC	*β* = 0.409 (0.294, 0.490), Δ*R* ^2^ = 0.154**	*β* = 0.315 (0.203, 0.410), Δ*R* ^2^ = 0.091**	*β* = 0.087 (−0.021, 0.166), Δ*R* ^2^ = 0.007
Dating/Being out late conflicts with FC	*β* = 0.402 (0.216, 0.369), Δ*R* ^2^ = 0.142**	*β* = 0.373 (0.196, 0.354), Δ*R* ^2^ = 0.122**	*β* = 0.208 (0.061, 0.204), Δ*R* ^2^ = 0.038**
Autonomy conflicts with MC	*β* = 0.413 (0.247, 0.417), Δ*R* ^2^ = 0.167**	*β* = 0.299 (0.149, 0.325), Δ*R* ^2^ = 0.088**	*β* = 0.314 (0.136, 0.284), Δ*R* ^2^ = 0.097**
Culture preference conflicts with MC	*β* = 0.430 (0.284, 0.469), Δ*R* ^2^ = 0.180**	*β* = 0.315 (0.177, 0.369), Δ*R* ^2^ = 0.097**	*β* = 0.166 (0.037, 0.206), Δ*R* ^2^ = 0.027*
Dating/Being out late conflicts with MC	*β* = 0.350 (0.158, 0.310), Δ*R* ^2^ = 0.111**	*β* = 0.297 (0.119, 0.272), Δ*R* ^2^ = 0.080**	*β* = 0.259 (0.079, 0.209), Δ*R* ^2^ = 0.061**

*Note*: Standard coefficients are reported above, with 95% confidence intervals reported in parentheses. All linear regressions above covaried for age, gender identity, generational status, current living situation with their caregiver, and perceived social status.

Abbreviations: FC, female caregiver; IAC, intergenerational acculturation conflict; MC, male caregiver; PB, perceived burdensomeness; TB, thwarted belongingness.

* *p* < 0.01.

** *p* < 0.001.

### The mediating role of TB and PB

Before running mediation analyses, we tested all assumptions needed for multiple mediation models (PROCESS Model 4, SPSS). All regression analyses covaried for young adults' age, gender identity, generational status, current living situation with their caregiver, and perceived socioeconomic status. Findings support significant linear associations between IAC categories (i.e., autonomy conflicts, cultural preference conflicts, and dating/being out late conflicts) with female and male caregivers and PB and with TB (see Table 2 for standardized coefficients). Similar significant associations were found for PB and TB and the suicide ideation frequency outcome (see Table 2 for standardized coefficients). Thus, all assumptions for statistical mediation analyses were met.

#### Autonomy conflict

**FIGURE 1:**
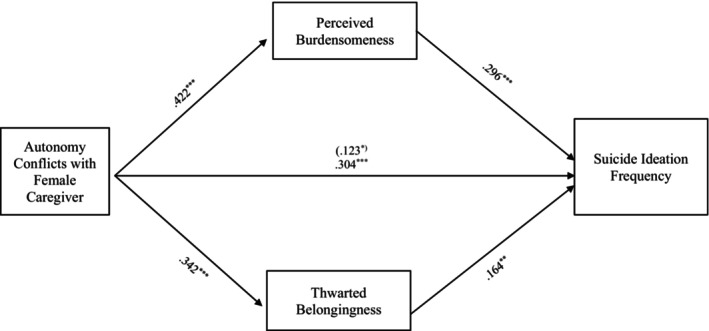
Autonomy conflicts with female caregiver mediation model. The relationship between autonomy conflicts with female caregiver and suicide ideation frequency was partially mediated by perceived burdensomeness and thwarted belongingness scores when controlling for age, gender identity, generational status, currently living with female caregiver status, and perceived socioeconomic status. The statistic within the parentheses is c’. Data represent standardized regression coefficients using 10,000 bootstrap samples to obtain bias‐corrected and accelerated 95% confidence intervals (*n* = 339). *p < 0.05; **p < 0.01; ***p < 0.001. Figures do not represent temporal relationships between variables, given the cross‐sectional nature of the data.

**FIGURE 2:**
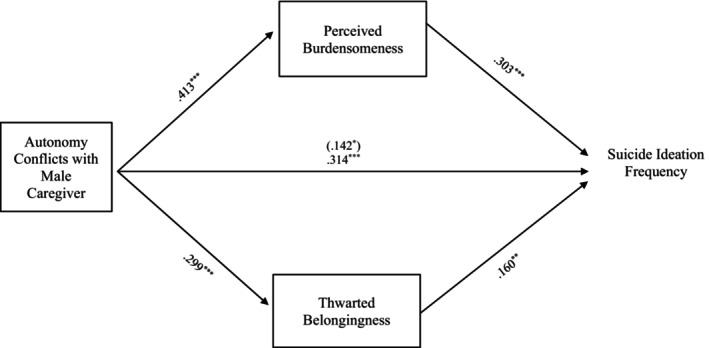
Autonomy conflicts with male caregiver mediation model. The relationship between autonomy conflicts with male caregiver and suicide ideation frequency was partially mediated by perceived burdensomeness and thwarted belongingness when controlling for age, gender identity, generational status, currently living with male caregiver status, and perceived socioeconomic status. The statistic within the parentheses is c’. Data represent standardized regression coefficients using 10,000 bootstrap samples to obtain bias‐corrected and accelerated 95% confidence intervals (*n* = 295). *p < 0.05; **p < 0.01; ***p < 0.001. Figures do not represent temporal relationships between variables, given the cross‐sectional nature of the data.

Results indicate that the entire model examining the relationship between suicide ideation frequency and autonomy conflict, through TB and PB, accounted for significant variance in the model with female caregivers, *R* ^2^ = 0.236, *F* (8, 330) = 12.718, *p* < 0.001, and in the model with male caregivers, *R* ^2^ = 0.234, *F* (8, 286) = 10.940, *p* < 0.001. The indirect effects of autonomy conflicts with the female caregivers to suicide ideation frequency through PB, *b = 0*.125, 95% CI: [0.059, 0.195], and TB, *b = 0*.056, 95% CI: [0.015, 0.105], were significant and support Hypothesis 3 (see Figure 1). Similarly, the indirect effects from autonomy conflicts with the male caregivers to suicide ideation frequency through PB, *b = 0*.125, 95% CI: [0.052, 0.204] and TB, *b = 0*.048, 95% CI: [0.008, 0.093] were significant and support Hypothesis 3 (see Figure 2).

#### Cultural preference conflict

**FIGURE 3:**
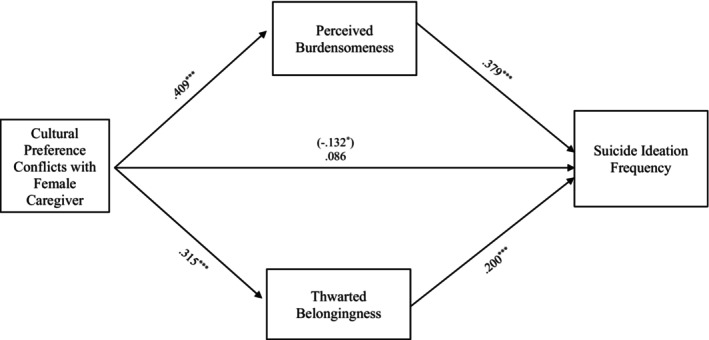
Cultural preference conflicts with female caregiver mediation model. The relationship between cultural preference conflicts with female caregiver and suicide ideation frequency was partially mediated by perceived burdensomeness and thwarted belongingness scores when controlling for age, gender identity, generational status, currently living with female caregiver status, and perceived socioeconomic status. The statistic within the parentheses is c’. Data represent standardized regression coefficients using 10,000 bootstrap samples to obtain bias‐corrected and accelerated 95% confidence intervals (*n* = 339). *p < 0.05; **p < 0.01; ***p < 0.001. Figures do not represent temporal relationships between variables, given the cross‐sectional nature of the data.

**FIGURE 4:**
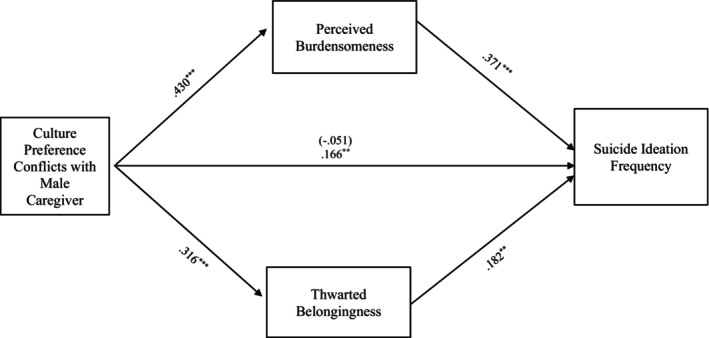
Cultural preference conflicts with male caregiver mediation model. The relationship between culture preference conflicts with female caregiver and suicide ideation frequency was partially mediated by perceived burdensomeness and thwarted belongingness scores when controlling for age, gender identity, generational status, currently living with male caregiver status, and perceived socioeconomic status. The statistic within the parentheses is c’. Data represent standardized regression coefficients using 10,000 bootstrap samples to obtain bias‐corrected and accelerated 95% confidence intervals (*n* = 295). *p < 0.05; **p < 0.01; ***p < 0.001. Figures do not represent temporal relationships between variables, given the cross‐sectional nature of the data.

The entire model examining the relationship between suicide ideation frequency and culture preference conflict through TB and PB also accounted for significant variance in the model with female caregivers, *R* ^2^ = 0.237, *F* (8, 330) = 12.820, *p* < 0.001, and in the model with male caregivers, *R* ^2^ = 0.220, *F* (8, 286) = 10.096, *p* < 0.001. More specifically, the indirect effects from cultural preference conflicts with the female caregiver to suicide ideation frequency through PB, *b = 0*.155, 95% CI: [0.091, 0.226] and TB, *b = 0*.063, 95% CI: [0.023, 0.110] were also significant and support Hypothesis 3 (see Figure 3). Similarly, the indirect effects from culture preference conflicts with the male caregiver to suicide ideation frequency through PB, *b = 0*.160, 95% CI: [0.083, 0.246] and TB, *b = 0*.057, 95% CI: [0.016, 0.104] were significant and support Hypothesis 3 (see Figure 4).

#### Dating/Being out late conflict

**FIGURE 5:**
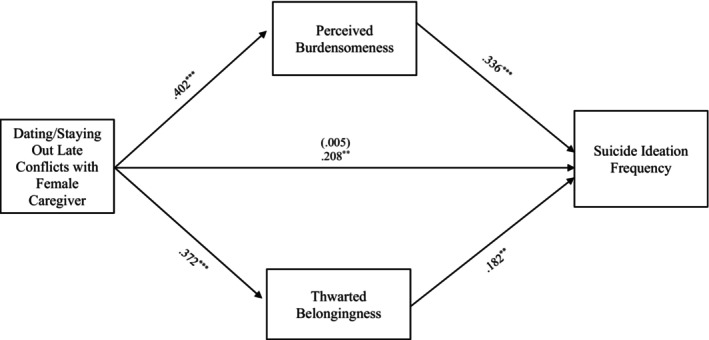
Dating/Staying out late conflicts with female caregiver mediation model. The relationship between dating/staying out late conflicts with female caregiver and suicide ideation frequency was partially mediated by perceived burdensomeness and thwarted belongingness scores when controlling for age, gender identity, generational status, currently living with female caregiver status, and perceived socioeconomic status. The statistic within the parentheses is c’. Data represent standardized regression coefficients using 10,000 bootstrap samples to obtain bias‐corrected and accelerated 95% confidence intervals (*n* = 364). *p < 0.05; **p < 0.01; ***p < 0.001. Figures do not represent temporal relationships between variables, given the cross‐sectional nature of the data.

**FIGURE 6:**
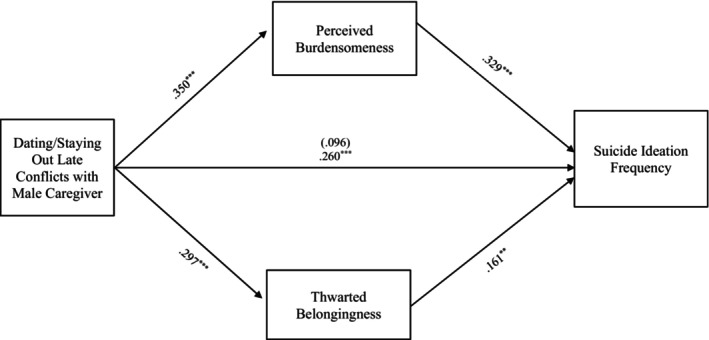
Dating/Staying out late conflicts with male caregiver mediation model. The relationship between dating/staying out late conflicts with male caregiver and suicide ideation frequency was partially mediated by perceived burdensomeness and thwarted belongingness scores when controlling for age, gender identity, generational status, currently living with male caregiver status, and perceived socioeconomic status. The statistic within the parentheses is c’. Data represent standardized regression coefficients using 10,000 bootstrap samples to obtain bias‐corrected and accelerated 95% confidence intervals (*n* = 295). *p < 0.05; **p < 0.01; ***p < 0.001. Figures do not represent temporal relationships between variables, given the cross‐sectional nature of the data.

The final full model examining the relationship between suicide ideation frequency and dating/being out late conflict through TB and PB also accounted for significant variance in the model with female caregivers, *R* ^2^ = 0.224, *F* (8, 330) = 11.896, *p* < 0.001, and male caregivers, *R* ^2^ = 0.226, *F* (8, 286) = 10.408, *p* < 0.001. Additionally, the indirect effects from dating/staying out late conflicts with the female caregiver to suicide ideation frequency through PB, *b = 0*.135, 95% CI: [0.069, 0.210] and TB, *b = 0*.068, 95% CI: [0.021, 0.127] were significant and support Hypothesis 3 (see Figure 5). Similarly, the indirect effects from dating/staying out late conflicts with the male caregiver to suicide ideation frequency through PB, *b = 0*.115, 95% CI: [0.052, 1.193] and TB, *b = 0*.048, 95% CI: [0.008, 0.100] were significant and support Hypothesis 3 (see Figure 6).

## DISCUSSION

The current study unites two bodies of literature, the IAC suicide risk literature, predominately studied among Latine and Asian/Asian American populations, and the IPTS literature, predominately studied among non‐Latine White populations. In doing so, this study not only (1) helps to examine the applicability of IPTS to a sample of Mexican descent emerging adult college students but also (2) helps to unpack the relationship between IAC and suicide ideation frequency by examining underlying pathways from the IPTS. In line with previous IAC literature, this study found significant relationships between greater levels of all categories of IAC (autonomy, cultural preference, dating/staying out late) and greater suicide ideation frequency, except for culture preference conflicts with female caregiver. Moreover, hypotheses positing that these relationships would be mediated through TB and PB were supported. Therefore, this study provides some evidence for generalizing the IPTS to Mexican descent college students. This study supports previous but limited literature examining the IPTS in Latine samples, which found links between PB and TB and suicidal desires (Acosta et al., 2017; Garza & Pettit, 2010). Given the collectivistic nature and essential social bonds, such as familism, common in Mexican communities, it makes sense that the IPTS, which considers social connection and interdependence, would apply to these populations (Almeida et al., 2009). Some research finds that Latine individuals in the United States, particularly Mexican foreign‐born individuals, rely on family ties for support more than non‐Latine White individuals (Almeida et al., 2009). However, given the particular importance of family among many US Mexican individuals, the link between IAC to TB and PB may be more significant compared to other social interactions and/or conflicts (i.e., college peers) not directly examined in the current study. Therefore, this study helps to extend the IPTS when examining IAC, and family conflict in particular, and should not be generalized across other social interactions or conflicts experienced by Mexican descent college students.

Study results also help tease out the pathways through which IAC is linked to suicide ideation frequency. In finding TB and PB to help explain in part the relationship between IAC categories to suicide ideation frequency, we can begin to understand *how* IAC functions. IAC itself may not directly explain increases in suicide ideation frequency. However, the pathways through which IAC leads to higher TB and PB may be vital to understanding the role of sociocultural factors on suicide ideation. As previously mentioned, familism is particularly important among Mexican individuals. Therefore, family conflicts with caregivers may make individuals feel like they are breaking cultural expectations. These feelings may be explicitly reinforced through caregivers or extended family. It is also possible that better effective communication, empathy, and perspective‐taking from caregivers and youth can mitigate the impact of IAC on TP and PB. As previously cited, some research has found a greater social connection with caregivers to buffer the impact of IAC on suicide (Piña‐Watson et al., 2019). Similarly, we used an IAC scale that was developed to include items endemic in acculturation‐related issues or topics of discussion that increase tension/disagreement and create unresolved conflicts between immigrant parents and young adults (Basáñez et al., 2014). Findings from this study provide more nuanced perspectives into what types of topics between immigrant parents and young adults may be driving distress, parent‐conflict, and suicide ideation among Latine college students. These findings have important implications for developing future culturally responsive suicide interventions for emerging adults, targeting improved belongingness and connectedness with caregivers to decrease conflicts arising from differences in acculturation.

Finally, this study continues to provide evidence for the importance of studying both male and female caregiver relationships with individuals of Mexican descent. The present study found consistent results in the associations in the models for both the male and female caregiver models, indicating that IAC with caregivers of both genders is associated with suicide outcomes. These results are consistent with Piña‐Watson et al. (2020), who also found associations between increased male and female caregiver IAC and suicide risk in a sample of Mexican descent adolescents and emerging adults.

### Limitations

These findings should be interpreted in light of a few study limitations. First, the study only examined Mexican descent emerging adults. Although this helped to not perpetuate the homogenization of all Latine individuals into a single category, the study is limited in generalizability to other Latine groups or ethnic/racial groups. Similarly, this study examined college students who may not have similar experiences to other Mexican descent non‐college attending peers. It is possible that emerging of Mexican descent adults not enrolled in college do not fit into the IPTS or support patterns found in the IAC literature. Another significant limitation is the cross‐sectional nature of our data. The parameter estimates derived from our cross‐sectional mediation model may overestimate and underestimate the relationships between our study variables (Maxwell & Cole, 2007). A longitudinal design could gather more discrete information to elucidate developmental and temporal patterns. The temporal relationships between factors and the directionality of predictors could be verified and/or strengthened with a longitudinal design.

Furthermore, given the low prevalence rate of suicide attempts in our sample (2%), it was not possible to examine suicide attempts in our analyses. Suicide attempt rates may have been underestimated in our study, given that we did not assess attempts separately from ideation (i.e., item used to assess suicide attempts asked participants if they “have ever thought about or attempted to kill yourself?”). However, our measure of suicide ideation provided essential insights into current prevalence rates of suicide ideation frequency among Mexican descent college students, such that 33.6% of the current sample reported experiencing suicidal ideation at least once in the last 12 months. Still, future research should employ more nuanced characterization of suicidal ideation outcomes by considering suicidal motivational and intentional states (Millner et al., 2023). Caregiver‐rated data was also not acquired. Instead, this study used youth perceptions of caregiver conflict, and the tested mediation models included different samples sizes because not all participants reported IAC for both female and male caregivers. Multi‐informant data can provide a more holistic understanding of the caregiver‐youth dynamic and allow for more fine‐grained dyadic analyses.

### Implications and future research

Some of the discussed limitations can serve as springboards for future research. First, examining the current study's hypotheses among a large ethnoracially diverse sample of Latine emerging adults of various educational backgrounds and their caregivers is necessary to replicate these findings. Additionally, extensive longitudinal studies with multiple time points are necessary to understand the developmental trajectories of suicide and to elucidate the mechanisms and predictive factors of suicide. Moreover, research has found that cultural factors such as collectivism, family cohesion, ethnic pride, and religiosity protect against suicide risk (Burnett‐Zeigler et al., 2013; Ruiz et al., 2013)—however, little research on how or if these cultural factors fit the IPTS remain unexamined. An important area of future research could be to examine how these protective factors fit in the models examined in this study. As previously stated, it is possible that IAC, in particular, is an important sociocultural factor that fits within the IPTS. Future studies should examine if friend/college peer social interactions/conflicts similarly link to suicide ideation frequency through TB and PB.

Additionally, researchers should test how familism values are associated with IAC in predicting suicide ideation. Similarly, the role of generation (i.e., first generation vs. fifth generation) should be examined in the context of IAC and suicide risk as a potential moderator of this relationship. Context for migration history, including if young adults migrated with their caregivers versus alone should be assessed when examining IAC. These future directions have important implications for formulating suicide prevention interventions and treatments. Results could help improve the quality of risk assessments and screening procedures to reduce suicidal behaviors and deaths.

### Conclusion

Suicide is the third leading cause of death among emerging adult college students (CDC, 2022). Despite nearly five decades of intervention development and efficacy testing, rates of suicide attempts, particularly for Latine youth, have not improved (Meza & Bath, 2020). In order to disrupt these elevated rates, research that examines culturally relevant risk and protective factors is urgently needed. This study is among the first to test the IPTS and cultural factors, like IAC, among a large sample of Latine college students. Our findings indicate that IAC with male and female caregivers is associated with suicide ideation frequency. This study found TB and PB to mediate the relationships between IAC and suicide ideation frequency. These findings advance our understanding of how sociocultural factors play a role in the IPTS predicting suicide risk and can inform the development of culturally responsive suicide interventions. Future treatment development for suicide should integrate skills to improve parent‐young adult communication around acculturation differences and should also focus on providing skills for coping with distress that stems from acculturation discrepancies with caregivers.

## CONFLICT OF INTEREST STATEMENT

The authors report no conflicts of interest.

## Data Availability

Research data for this study are not shared.
